# Higher Masticatory Performance and Higher Number of Chewing Strokes Increase Retronasal Aroma

**DOI:** 10.3389/fnut.2021.623507

**Published:** 2021-03-02

**Authors:** Jumpei Okawa, Kazuhiro Hori, Tasuku Yoshimoto, Simonne E. Salazar, Takahiro Ono

**Affiliations:** Division of Comprehensive Prosthodontics, Niigata University Graduate School of Medical and Dental Sciences, Niigata, Japan

**Keywords:** retronasal aroma, masticatory performance, chewing strokes, swallowing threshold, stimulated salivary flow

## Abstract

Mastication is a physiological process whereby food is comminuted and mixed with saliva to form a swallowable bolus; it is also the initial process for retronasal aroma that is released from foods to receptors in the nose. However, the influence of mastication state on retronasal aroma is poorly understood. The purpose of this study was to investigate the relationship between aroma concentration and factors related to mastication state. The study design was an analytical observational study. Twelve male volunteers (age, 26.5 ± 2.7 years) were recruited and divided into five and seven participants in the low and high masticatory performance groups, respectively. The stimulated salivary flow rate was measured while participants chewed paraffin wax. First, an odor sensor was placed in the nostril, and the aroma concentration was measured over time as participants chewed an orange-flavored gummy jelly standardized for masticatory performance assessment until swallowing; chewing strokes were counted to determine swallowing thresholds. Next, participants were instructed to chew the gummy jelly for a certain number of strokes (i.e., 50 or 100% of swallowing thresholds, as well as 30 strokes) and expectorate the jelly without swallowing. The surface area of comminuted jelly at 30 chewing strokes was defined as masticatory performance. Maximum and slope of aroma concentration, surface area, number of chewing strokes, and stimulated salivary flow rate were compared between low and high masticatory performance groups. Statistical significance was set at α = 0.05. At 30 chewing strokes, the maximum aroma concentration and the slope were significantly greater in the high masticatory performance group than in the low masticatory performance group. There was a positive correlation between the maximum aroma concentration and the number of chewing strokes with aroma release in both groups. No significant correlation was found between the maximum aroma concentration and the stimulated salivary flow rate. However, multiple regression analysis (with aroma concentration as a dependent variable) showed that the increase in surface area, the number of chewing strokes, and the stimulated salivary flow rate were significant explanatory variables. The results suggested that retronasal aroma was influenced by mastication state and salivary flow rate during chewing.

## Introduction

Mastication is a physiological process whereby food is comminuted, mixed with saliva, and formed into a bolus that can be swallowed safely (1). Mastication also serves to release aromas from foods, which are transported through the pharynx to the nose and perceived (these comprise retronasal aromas). Masticatory function and retronasal perception during food intake have been studied in relation to eating behavior that leads to metabolic syndrome and obesity (2–6). However, the influence of an individual's physiological mastication state (i.e., mastication condition) on retronasal aroma is poorly understood.

The aromas from foods comprise volatile compounds that are released from the food surface by diffusion between food and air phases (7–9). The surface area between these phases is formed as a result of food comminution by chewing (10) and is increased by a large number of chewing strokes (11, 12). Masticatory performance is an objective parameter for evaluating the state of food comminution by chewing; high masticatory performance enables comminution of foods more efficiently with a small number of chewing strokes (12). Tarrega et al. investigated the aroma released from cheeses with the chewing activity (13) and the masticatory performance measured using a silicone rubber (14); however, the aroma release was related to the chewing behavior, but relationship with masticatory performance was not found. In recent years, masticatory performance has been assessed by measuring the increase in surface area of standardized and flavored gummy jelly after 30 chewing strokes (15–17); this parameter is reportedly associated with metabolic syndrome (4).

Furthermore, the properties of food boluses are influenced by the mixing with saliva (18, 19). Saliva is secreted by stimulation during chewing, which facilitates food bolus formation (20). A reduction in stimulated salivary flow rate reduces masticatory performance (21). In gelatin-based foods such as the gummy jelly, the reduction in food hardness caused by saliva leads to increased aroma release (8). However, the aroma dissolves in saliva and is thus attenuated (22). Feron et al. (23) reported that the stimulated salivary flow rate is negatively correlated with aroma release during mastication.

During the progression of mastication, a swallowable food bolus is formed until the swallowing reflex is triggered; this constitutes the swallowing threshold (12, 24). The masticatory performance and stimulated salivary flow rate also influence the number of chewing strokes until the swallowing threshold is reached (25).

Therefore, physiological parameters such as masticatory performance, number of chewing strokes, and salivary flow rate interact with each other and may be associated with retronasal aroma. We constructed the following hypothesis regarding the physiological parameters that might influence retronasal aroma release while chewing the gummy jelly: (1) higher masticatory performance and more chewing strokes lead to increased retronasal aroma and (2) high stimulated salivary flow rate reduces retronasal aroma. To test this hypothesis, we measured the aroma concentration (through the nostril) over time while chewing the gummy jelly in participants with different levels of masticatory performance. This study was performed to clarify differences in retronasal aroma release depending on masticatory performance and to investigate the relationships of aroma concentration with masticatory performance, number of chewing strokes, and stimulated salivary flow rate.

## Materials and Methods

### Participants

The participants in this study were 12 male volunteers (age, 26.5 ± 2.7 years; body mass index, min–max = 17.8–30.9) who had complete dentition; none had a history of dysphagia, neuromuscular disorders, chronic sinus infection (nasal obstruction), respiratory diseases, temporomandibular disease, taste and smell disorder, or dry mouth. This study conformed to the standards of the Declaration of Helsinki and was approved by the Ethics Committee of Niigata University Faculty of Dentistry (23-R35-03-01). All volunteers provided written informed consent to participate in this study.

### Test Samples

A gummy jelly (5.5 g, 20 × 20 × 10 mm, UHA Mikakuto Co., Ltd., Osaka, Japan), which had been standardized for masticatory performance assessment, was used as a test food (15–17). The main component of the jelly was gelatin; moreover, the jelly contained orange flavor and the β-carotene pigment. Paraffin wax without any taste or odor (1.0 g, OralCare. Inc, Tokyo, Japan) was used for measurement of stimulated salivary flow rate.

### Equipment

#### Odor Sensor

During chewing, aroma concentration was measured over time using a portable odor sensor (XP-329IIIR; New Cosmos Electric Co., Ltd., Tokyo, Japan). The odor sensor absorbed at a suction flow rate of 450 ± 150 ml/min and output aroma concentration over time; it was equipped with indium oxide-based high-sensitivity hot-wire semiconductor sensors. When the absorbed odor compounds adhered to the sensors, the resistance level was reduced. This change in resistance level was used as the output voltage deviation of the bridge; it was converted to a numerical representation of aroma concentration over time. Because the sensor specificity was not limited to particular aromatic compounds, multicompound odors could be detected. The output aroma concentration was recorded as a relative value in relation to a calibration odor without any units. The calibration odor was generated when the aromatic compound gas adhered to and was removed by a built-in active carbon filter in the device. The sampling frequency was 2 Hz. The data output from the odor sensor were recorded by the computer in real time using MATLAB version R2016b (The MathWorks Inc., Natick, MA, USA).

#### Masticatory Performance Measurement

Masticatory performance was evaluated based on the increase in surface area of the comminuted gummy jelly using a smartphone (SH-M05, Android version 8.0.0, Sharp Corporation, Osaka, Japan) (17). This is a programming designed based on the previous method (15, 16) for evaluating the increase in surface area from the elution of β-carotene. The comminuted gummy jelly was washed with water and then transferred to a dedicated box containing 30 ml of water. The increase in surface area of the comminuted gummy jelly was calculated using a photograph taken by a smartphone camera without flash, following image processing (17).

#### Electromyography

The muscle activities of masseter and suprahyoid muscles during chewing were recorded using a wireless surface electromyography system (Trigno Wireless EMG System; Delsys Inc., Natick, MA, USA), which uses an active electrode with a fixed inter-electrode distance of 10 mm. The electrodes were placed in the left and right masseter muscles and the suprahyoid muscle group; the positions were determined by muscle palpation. The sampling frequency was 1 kHz.

#### Throat Microphone

The swallowing sound was measured by a throat microphone (SH-12jk, NANZU ELECTRIC Co., Ltd., Sizuoka, Japan), which was placed in the neck region. The sampling frequency was 40 kHz.

#### Synchronizing System

Electromyography activity and sound were recorded with an analog-to-digital converter (Power Lab, AD Instruments, New South Wales, Australia). A synchronizing signal was recorded in both PowerLab and MATLAB, then used to synchronize all data. The stored data in PowerLab were imported to MATLAB, and the time axes were matched based on the synchronizing signal.

### Data Collection

The test for stimulated salivary flow rate was carried out once before measurement of aroma concentration to prevent stimulation from chewing the gummy jelly. Before saliva collection, each participant chewed the paraffin wax until it became soft, then swallowed the saliva in the oral cavity. Beginning at that point, the participant chewed the paraffin wax continuously for 5 min. The saliva secreted during the measurement was expectorated into a measuring cup at short intervals (26).

Next, the participant was asked to sit in a chair. An odor sensor with a 15-cm nasal tube (Nasal Cannula Ref#302-E; 2-mm inner diameter; Unomedical, Inc., McAllen, TX, USA) attached to the nose piece was placed in the participant's both nostrils (27).

In the present study, the following two experiments were conducted to measure aroma concentration during chewing:

Experiment (1) Aroma concentration during free intake. Each participant was instructed to chew and swallow the gummy jelly freely. The aroma concentration was measured from the start of chewing the gummy jelly until the first swallowing. The number of chewing strokes before swallowing was recorded as the swallowing threshold.

Experiment (2) Aroma concentration and increase in surface area for a certain number of chewing strokes. The numbers of chewing strokes were set to 50 and 100% of swallowing thresholds, as well as 30 strokes. The aroma concentration was measured from the start of chewing the gummy jelly until the designated number of chewing strokes was reached. Then, the comminuted gummy jelly was expectorated without swallowing, and the increase in surface area was calculated by image analysis.

To calibrate aroma concentration, each participant was instructed to place the gummy jelly in the oral cavity 15 s before the start of each measurement and to breathe through the nose without chewing. A previous investigation confirmed that no aroma was released in this situation (27). The average aroma concentration during calibration was set as the baseline. After each measurement, participants were asked to rinse their mouths repeatedly with 5 ml of water and to swallow the water to clear the pharynx, then to wait until the aroma concentration decreased to baseline levels. Each of these four tasks (experiments 1 and 2) was performed three times, for total of 12 trials. In experiment 2, the order of tasks was chosen at random.

### Data Analysis

#### Increase in Surface Area of Comminuted Gummy Jelly and Masticatory Performance

For each participant, the average increase in gummy jelly surface area was calculated in each task; the masticatory performance was defined as the average value after chewing 30 strokes. The masticatory performance of each participant was evaluated with reference to the methods used by Kosaka et al. (28) and Nokubi et al. (29). Participants were divided into two groups: high masticatory performance (high MP group) and low masticatory performance (low MP group), using a cutoff value of 5,825 mm^2^ from previous studies (28, 29).

#### Stimulated Salivary Flow Rate

For each participant, the stimulated salivary flow rate was calculated as the volume of saliva expectorated during chewing, in milliliters per minute.

#### Aroma Concentration

**Figure 1:**
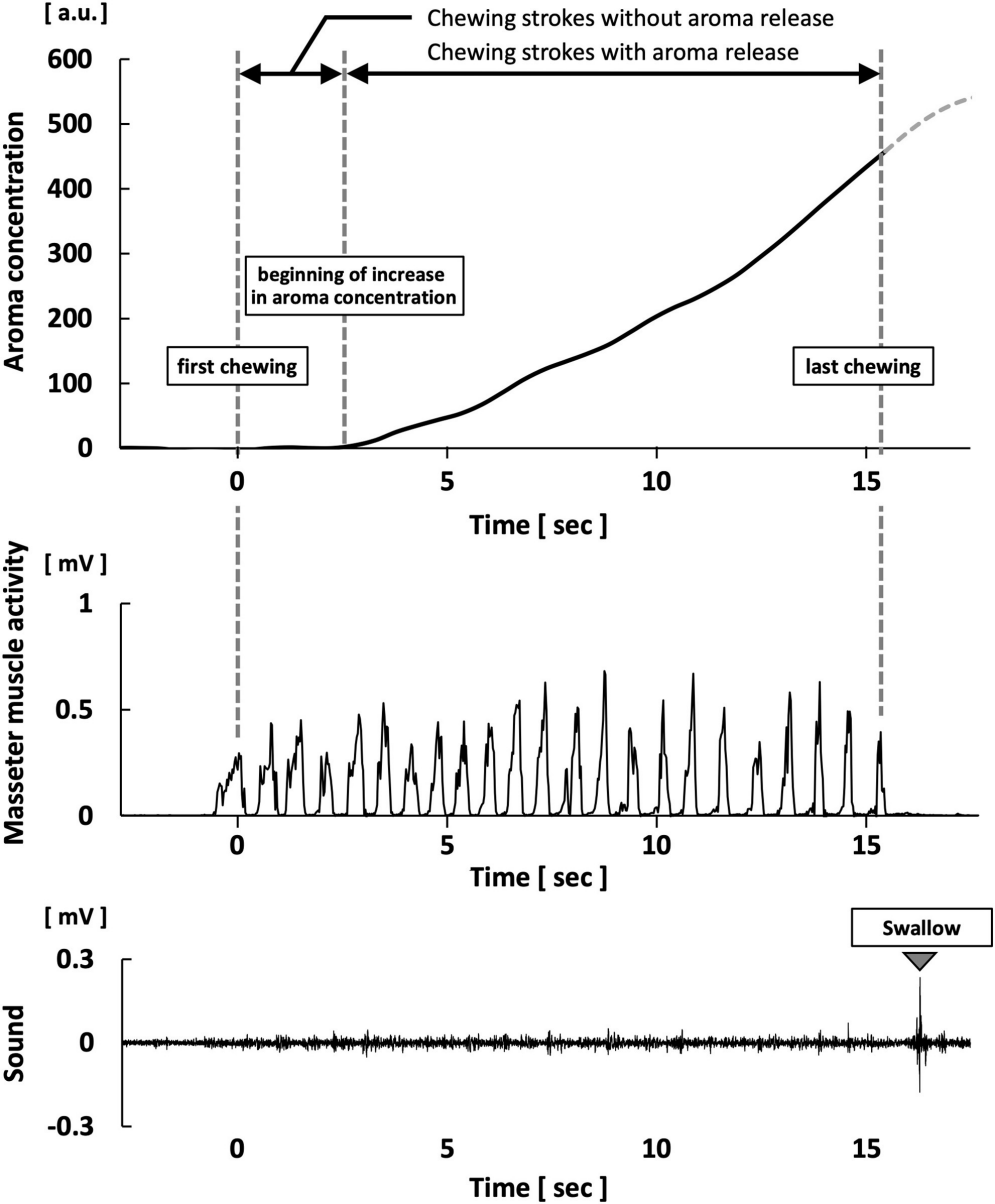
Waveforms of aroma concentration, masseter muscle activity, and swallowing sound during gummy jelly intake.

In preparation for the analysis, the measured aroma concentration was subtracted from the baseline level in the raw data; this was used as the calibrated aroma concentration. The maximum aroma concentration during chewing was calculated from the aroma concentration after calibration. The average slope was calculated by dividing the maximum aroma concentration by the duration of chewing strokes with aroma release; this was measured in aroma concentration *per second* (Figure 1).

#### Chewing Strokes

The electromyography waveform of the masseter muscle was converted to a full-wave rectified waveform; the root mean square (with a window length of 100 ms) was calculated for smoothing. The local maximum of the waveform was determined by software analysis as the masseter muscle burst due to chewing. The number and position of bursts during chewing were recorded as the number of chewing strokes and the timing of chewing, respectively (30). The swallowing was identified from the sound and the electromyographic waveform of the suprahyoid muscles. The swallowing threshold was defined as the number of chewing strokes during the period from the start of chewing (before the first swallow) and was averaged three times for each participant. During free intake, a calibration period of 15 s was recorded; then, the number of chewing strokes without aroma release was counted from the start of chewing to the onset of increasing aroma concentration.

### Statistical Analysis

The sample size was calculated from preliminary experiment data with α = 0.05 and detection power = 0.8. The numbers of participants in the low and the high MP group were 2 and 3. The variance normality and equality were examined in the low and high MP groups for each of the following parameters: increase in surface area of gummy jelly, the number of chewing strokes, and the aroma concentration. When variance normality and equality were present, Student's *t*-test was used for further analysis. When variance normality and equality were absent, the Mann–Whitney *U*-test was used for further analysis. Correlations were calculated using either the Pearson correlation coefficient or the Spearman rank correlation coefficient. The correlation between the number of chewing strokes and the aroma concentration, which were data for each measurement in Experiment 2, was calculated within-participant and within-group. The correlation between the increase in surface area of gummy jelly and the aroma concentration, which were averaged three times for each participant in Experiment 2 was were also calculated. Furthermore, multiple regression analysis with the forward-backward stepwise selection method was performed; aroma concentration was the dependent variable, while masticatory performance, number of chewing strokes, and stimulated salivary flow rate were independent variables. Statistical significance was set at α = 0.05. All statistical analyses were performed with IBM SPSS Statistics, version 23.0j (IBM Japan, Ltd., Tokyo, Japan).

## Results

### Masticatory Performance

**Figure 2:**
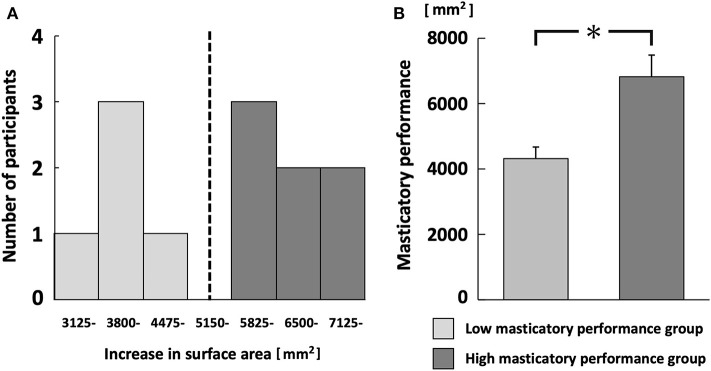
Histogram of masticatory performance **(A)** and comparison between low and high masticatory performance groups **(B)**. Black dotted line in **(A)** separates low and high masticatory performance groups. *p < 0.05.

There were five and seven participants in the low and high MP groups, respectively. The mean masticatory performances were 6,823 ± 715 mm^2^ (range, 5,927–7,748 mm^2^), 4,319 ± 396 mm^2^ (range, 3,757–4,858 mm^2^), respectively (*p* < 0.001; Figures 2A,B). There was a weak correlation between masticatory performance and stimulated salivary flow rate, but this was not statistically significant (*r* = 0.40, *p* = 0.20).

### Swallowing Threshold

**Figure 3:**
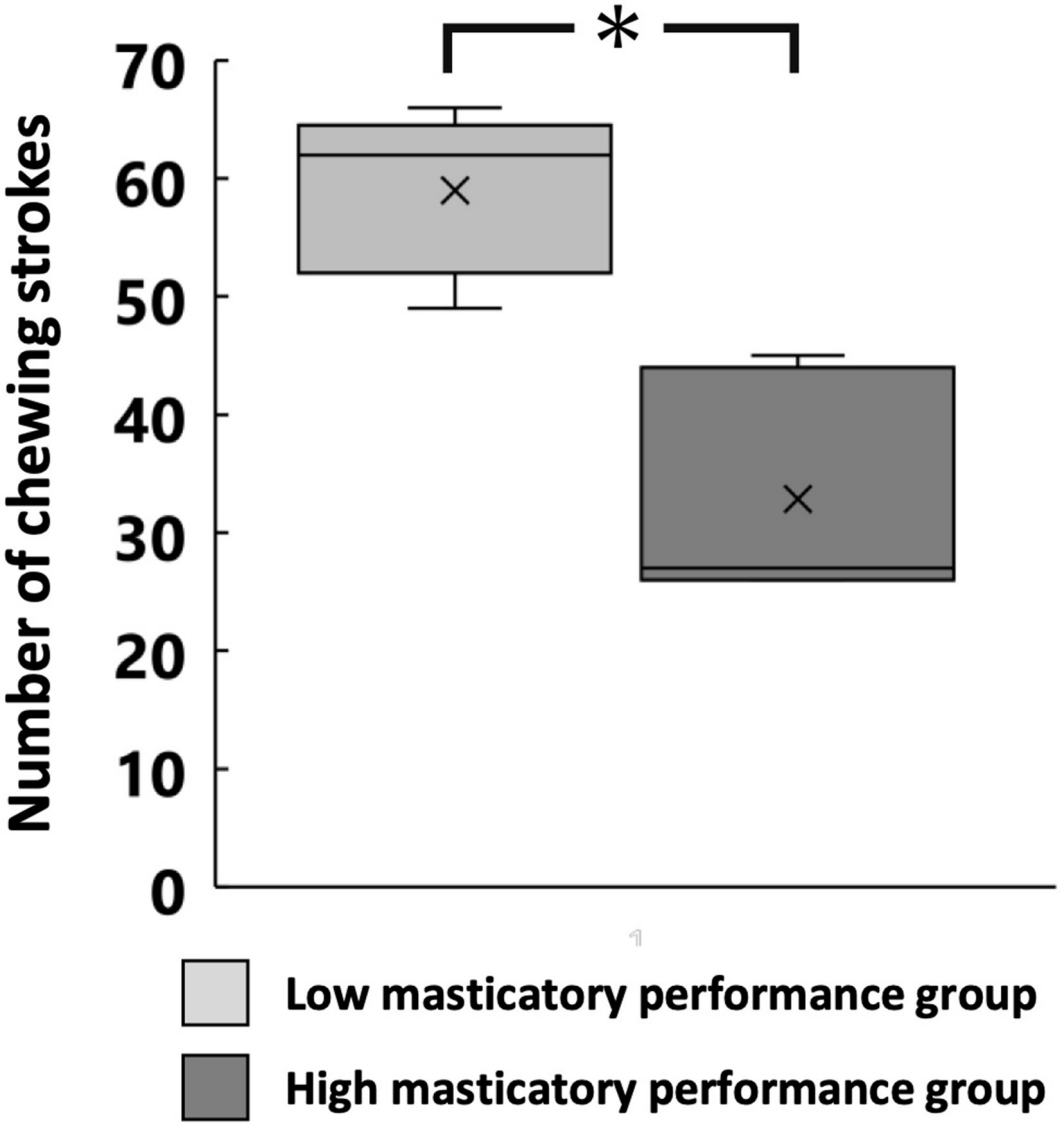
Number of chewing strokes at swallowing threshold for each group. *p < 0.05.

The swallowing threshold was significantly lower in the high MP group than in the low MP group (32.7 ± 8.2 and 59.0 ± 6.2, respectively, *p* < 0.05; Figure 3).

### Aroma Concentration During Free Intake

**Figure 4:**
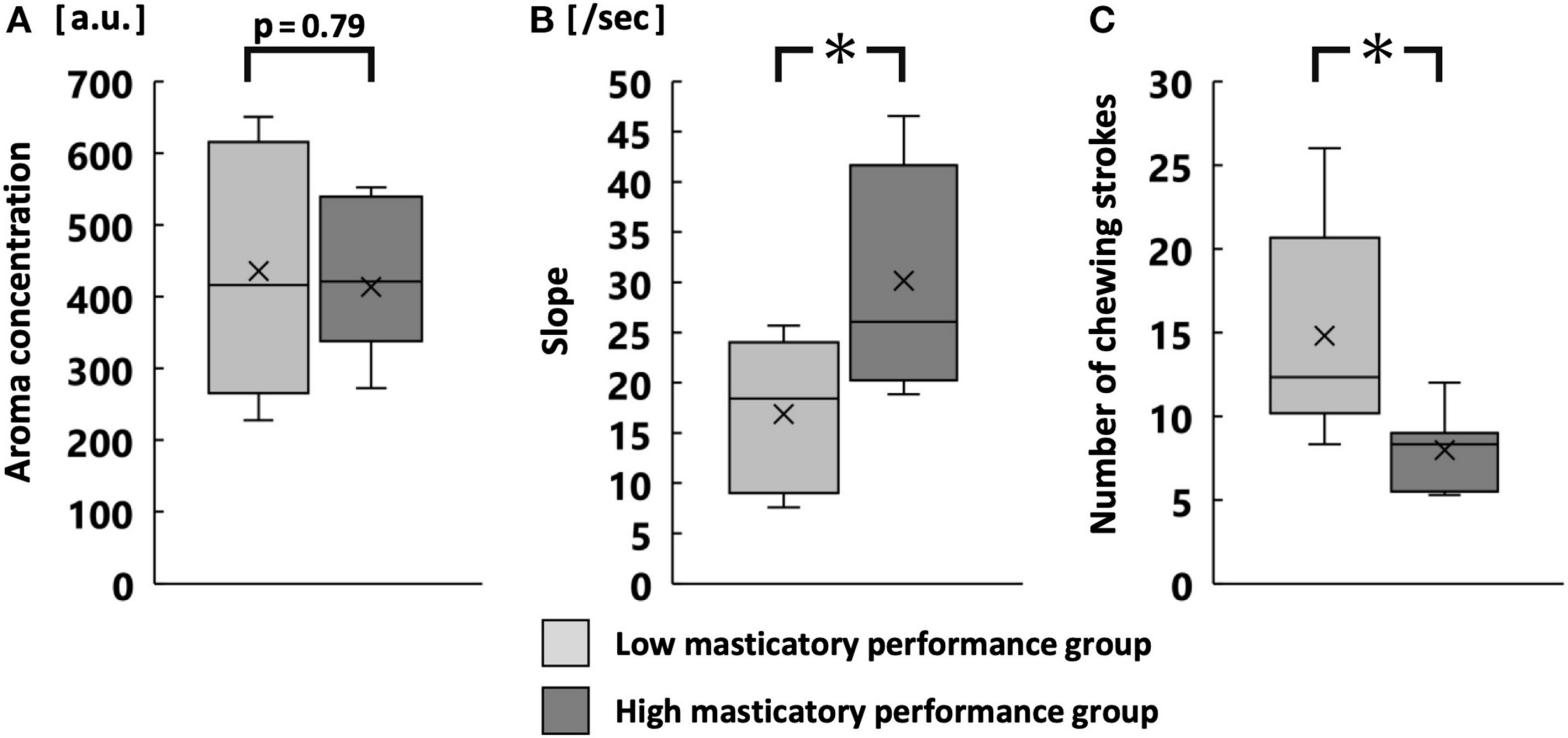
Maximum aroma concentration **(A)**, slope **(B)**, and number of chewing strokes without aroma release **(C)** during free intake. *p < 0.05.

There was no significant difference in the maximum aroma concentration during free intake (Figure 4A). However, the average slope was significantly greater in the high MP group than in the low MP group (*p* < 0.05; Figure 4B). The number of chewing strokes without aroma release was significantly smaller in the high MP group than in the low MP group (*p* < 0.05; Figure 4C). Supplementary Figure 1 shows averaged time-release plots of the aroma concentration for both groups.

### Aroma Concentration for a Specific Number of Chewing Strokes

**Figure 5:**
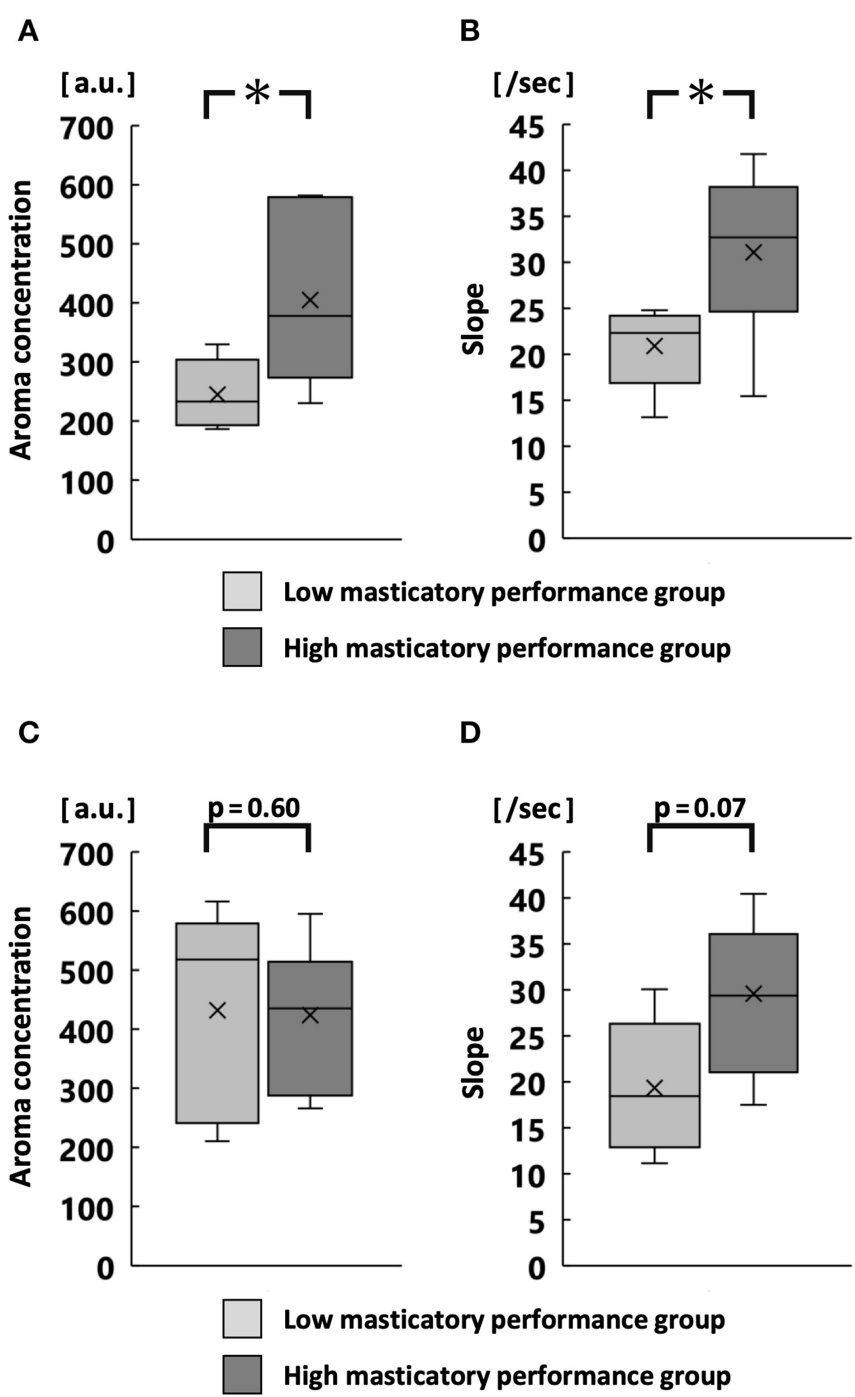
Maximum aroma concentration and slope at 30 strokes **(A,B)** and at chewing strokes equal to 100% of swallowing threshold **(C,D)**. *p < 0.05.

At 30 chewing strokes, the maximum aroma concentration and average slope were significantly greater in the high MP group than in the low MP group (*p* < 0.05; Figures 5A,B). At chewing strokes equal to 100% of the swallowing threshold, there was no significant difference in the maximum aroma concentration and the average slope (Figures 5C,D).

**Figure 6:**
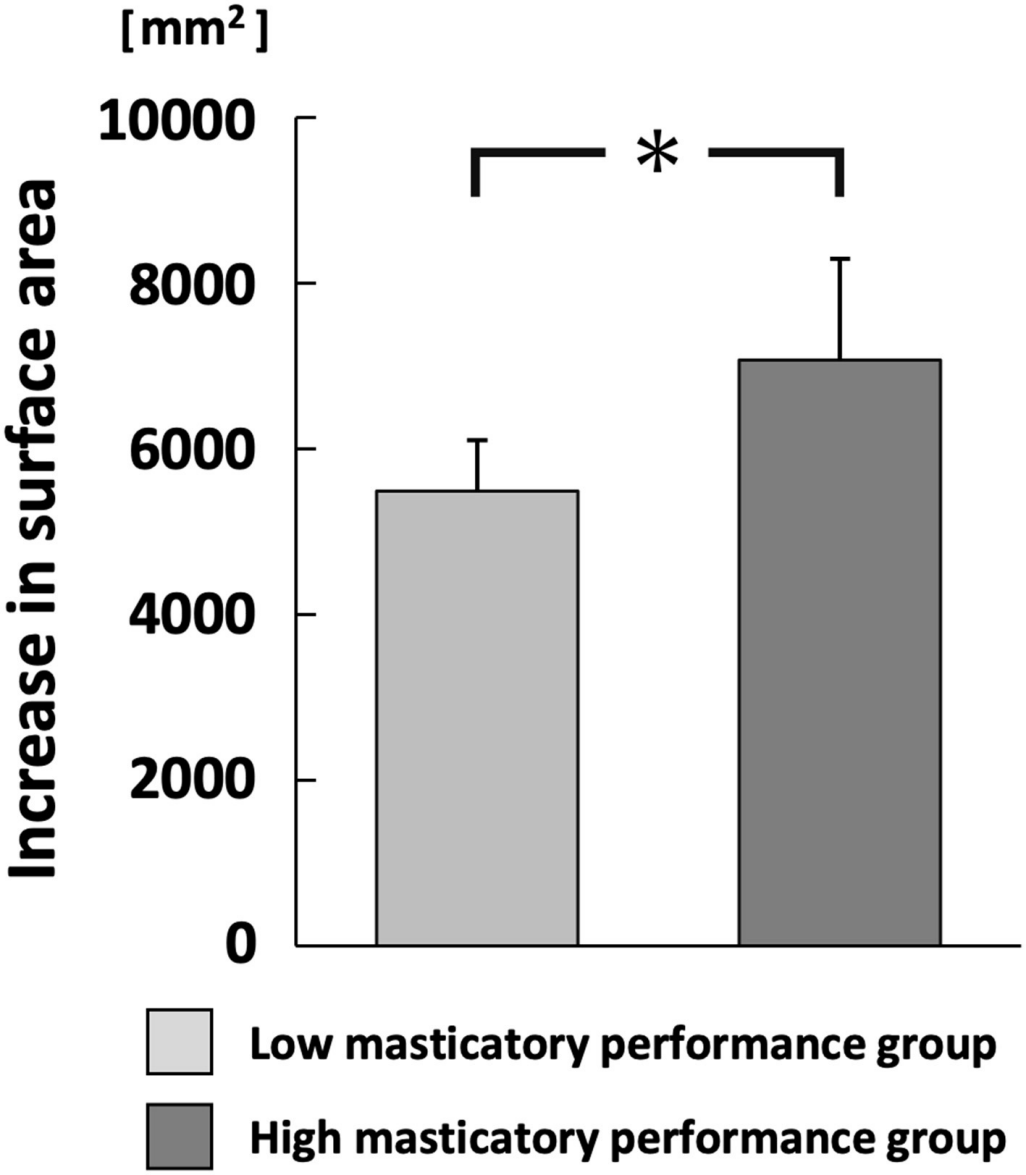
Increase in the surface area of comminuted gummy jelly at chewing strokes equal to 100% of swallowing threshold. *p < 0.05.

**Figure 7:**
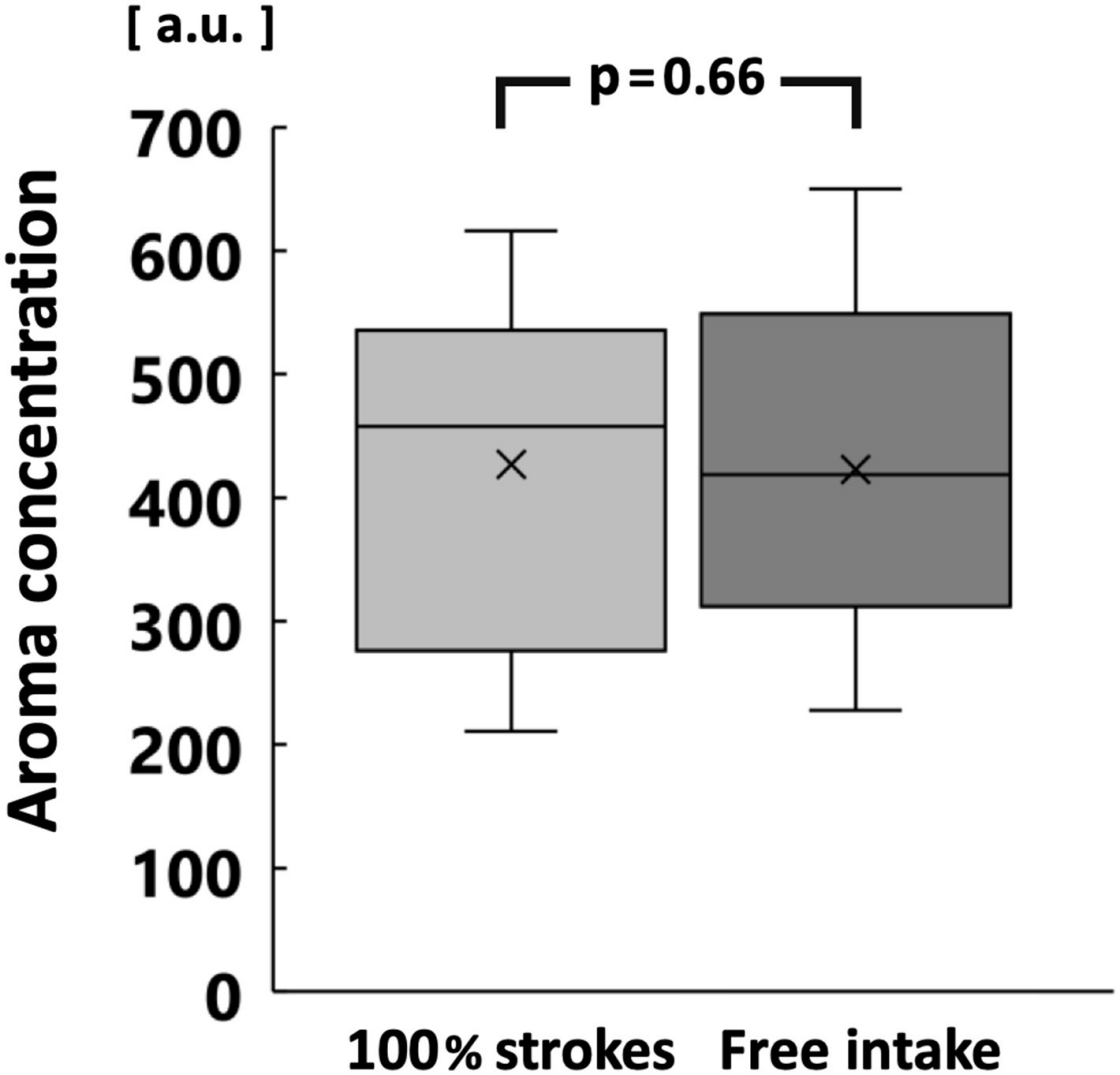
Comparison of maximum aroma concentration between chewing strokes equal to 100% of swallowing threshold and free intake. No significant difference was observed.

The increase in surface area of comminuted gummy jelly at chewing strokes equal to 100% of the swallowing threshold was significantly greater in the high MP group than in the low MP group (*p* < 0.05; Figure 6). There was no significant difference in the maximum aroma concentration between chewing strokes equal to 100% of the swallowing threshold and free intake (Figure 7).

### Correlations of Aroma Concentration With Chewing Strokes, Surface Area, and Stimulated Salivary Flow Rate

**Figure 8:**
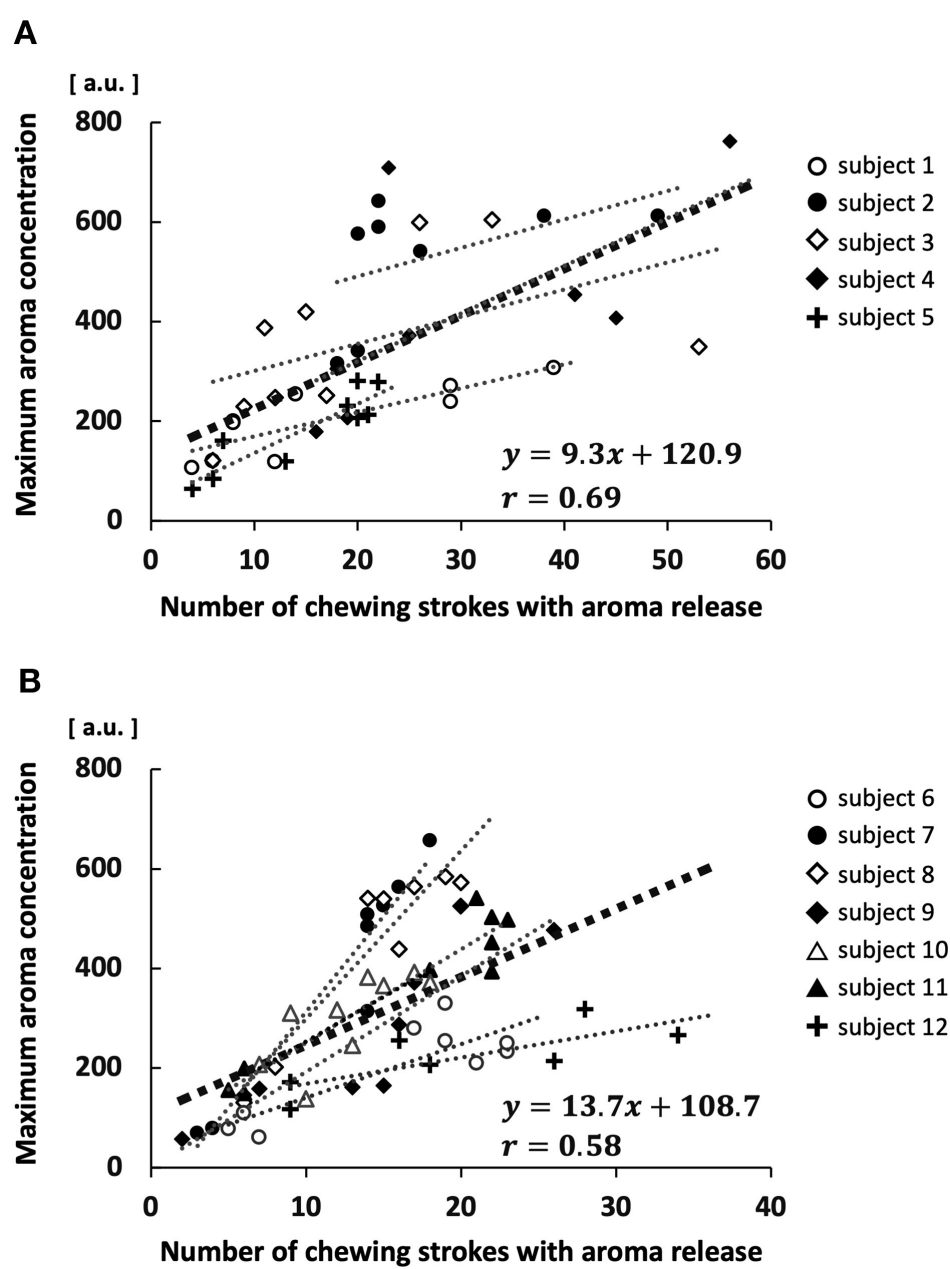
Correlation between maximum aroma concentration and number of chewing strokes with aroma release in each participant. **(A)** High masticatory performance group. **(B)** Low masticatory performance group.

There was a positive correlation between the maximum aroma concentration and the number of chewing strokes with aroma release in both the low MP group (*r* = 0.69, *p* < 0.01; Figure 8A) and high MP group (*r* = 0.58, *p* < 0.01; Figure 8B). This positive correlation was also observed in each participant; the average value of the correlation coefficient in each participant was 0.80 ± 0.13. Furthermore, the slope of linear regression was significantly greater in the high MP group (19.6 ± 9.9) than in the low MP group (7.2 ± 1.9; *p* < 0.05).

**Figure 9:**
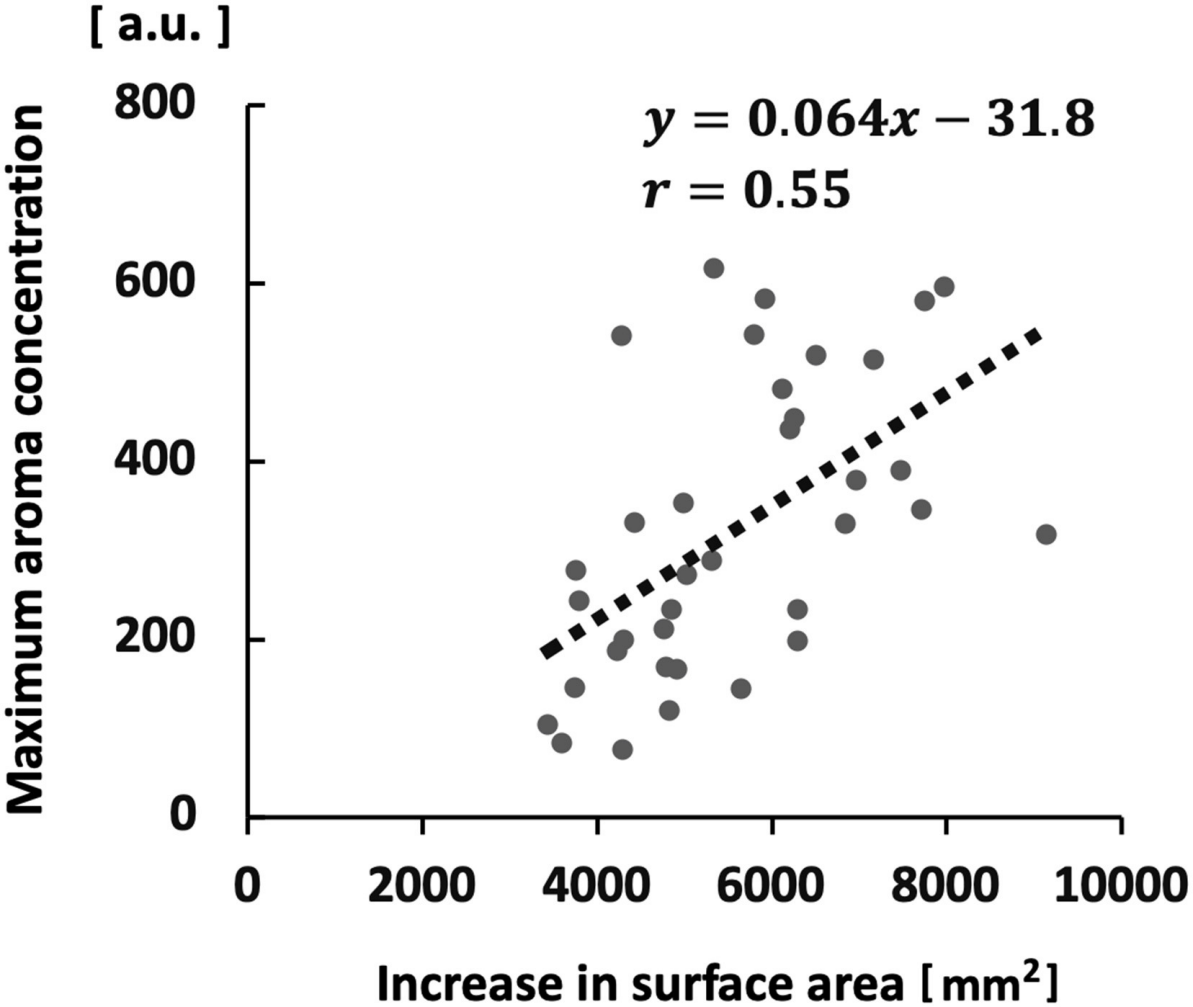
Correlation between maximum aroma concentration and increase in surface area.

A significant positive correlation was observed between the maximum aroma concentration and the increase in surface area (*r* = 0.55; *p* < 0.01; Figure 9). No significant correlation was found between the maximum aroma concentration and stimulated salivary flow rate.

### Multiple Regression Analysis

Table 1 shows the results of multiple regression analysis; aroma concentration was the dependent variable, while masticatory performance, number of chewing strokes, and stimulated salivary flow rate were independent variables. Masticatory performance, number of chewing strokes, and stimulated salivary flow rate were all identified as significant factors (*R*^2^ = 0.44).

**Table 1:** Multiple regression analysis of aroma concentration.

**Independent variables**	**B**	**β**	**SE**	***p***	**95% CI**	
(Intercept)	2.96		52.25	0.96	−100.70	106.61
Increase in surface area	0.04	0.36	0.01	<0.001	0.02	0.06
Number of chewing strokes	6.36	0.46	1.11	<0.001	4.15	8.56
Stimulated salivary flow rate	−40.74	−0.31	10.71	<0.001	−61.98	−19.49

*R^2^ = 0.44*.

*CI, confidence interval; SE, standard error*.

*Dependent variable is aroma concentration*.

## Discussion

The present study investigated the dynamics of retronasal aroma during the oral processing of gummy jelly in various physiological mastication states. The results showed that the aroma concentration (measured through the nostril) differed depending on masticatory performance and the number of chewing strokes. Furthermore, multiple regression analysis suggested that aroma concentration during food intake may be related to the increase in surface area of comminuted jelly, the number of chewing strokes, and the stimulated salivary flow rate.

### Methodological Considerations

The aroma concentration and masseter muscle activity were measured simultaneously during gummy jelly chewing, while masticatory performance was measured by examination of chewed gummy jelly. These methodologies allowed investigation of the relationship between retronasal aroma and physiological mastication state. Previous studies had some limitations in measuring masticatory function and aroma release measurements: masticatory function was measured by indirect evaluation such as electromyography and occlusal force tests, or aroma and masticatory function were measured by different tasks. In this study, masticatory performance was used to evaluate masticatory function, which is a quantitative and direct evaluation of measuring the comminuted sample and is a common technique in the dental field. Electromyography and occlusal force tests are parameters for estimating masticatory function, however they are indirect evaluation for investigating the state of muscles. Therefore, the masticatory performance using gummy jelly was adopted, which can directly and simultaneously evaluate the masticatory function that occurs as an operation of the state of muscles.

Chewing strokes equal to 100% of the swallowing threshold was used because mastication that exceeds the swallowing threshold requires swallowing suppression. The swallowing threshold varies greatly among individuals (12, 25); the present study also showed different swallowing thresholds depending on masticatory performance. Therefore, by setting the chewing strokes below the swallowing threshold for each participant, measurement during physiological mastication could be ensured; 30 strokes is an established number for assessment of masticatory performance using the gummy jelly (4, 21). The average swallowing threshold for the high MP group in this study was 32.7, which was not considerably different from 30.

### Masticatory Performance and Swallowing Threshold

The number of chewing strokes at swallowing threshold was lower in the high MP group than in the low MP group. In both groups, the food was crushed by efficient chewing and quickly formed into a swallowable bolus. However, the low MP group showed a small increase in the surface area of gummy jelly expectorated after chewing strokes equal to 100% of the swallowing threshold, although the number of chewing strokes was greater than in the high MP group. Participants with low masticatory performance increase the number of chewing stroke at the swallowing threshold, however they are not always able to comminuted the bolus into small pieces. These results were similar to the findings reported by Fontijn-Tekamp et al. (12).

### Relationships of Aroma Concentration With Masticatory Performance and Chewing Strokes

The slope of aroma concentration was significantly greater in the high MP group than in the low MP group. In model mouth systems, fast crushing foods are increase aroma release (31, 32). In this study, because the high MP group had a high food crushing efficiency, the area of aroma release rapidly increases, which increases the slope size. Therefore, the maximum aroma concentration was higher in the high MP group than in the low MP group after 30 strokes.

In contrast, there was no significant difference in maximum aroma concentration between groups during free intake or when using chewing strokes equal to 100% of the swallowing threshold. The low MP group had a higher number of chewing strokes equal to 100% of the swallowing threshold, compared with the high MP group. Large number of chewing stroke and long chewing duration are reportedly increase aroma intensity (13, 33). However, the increase in surface area of the gummy jelly at chewing strokes equal to 100% of the swallowing threshold was significantly smaller in the low MP group than in the high MP group. Therefore, for both reasons, there was no difference in maximum aroma concentration between groups. These findings imply that both the increase in surface area and the number of chewing strokes influence the aroma concentration. Our multiple regression analysis supported these findings.

### Chewing Strokes Without Aroma Release

Aroma concentration was not detected at the start of chewing the gummy jelly. During free intake, the number of chewing strokes in this period was lower in the high MP group than in the low MP group. Participants were required to expend considerable effort to chew the gummy jelly because of its high hardness. Therefore, the gummy jelly was not comminuted sufficiently during early mastication; sufficient surface area for aroma release could not be generated. In addition, respiratory rhythm is reportedly perturbed in the early stage of chewing solid food (34), while an increase in airway resistance has been observed due to narrowing of the velo-pharynx isthmus in soft palate elevation during chewing (35). The high MP group might have been able to accomplish the initial gummy jelly crushing with high-effort chewing by using a smaller number of chewing strokes, thus initiating early aroma release.

### Relationship Between Aroma Concentration and Stimulated Salivary Flow Rate

Masticatory performance tended to increase as the stimulated salivary flow rate increased, but there was no significant correlation between these parameters. There was also no significant correlation between the maximum aroma concentration and the stimulated salivary flow rate. Saliva changes the physical properties of gelatin, which is the main content of the gummy jelly; this leads to increased aroma release (8). However, Odake et al. (22) reported that the aroma component dissolves in saliva, resulting in a reduced aroma concentration and diminished aroma release. Saliva has both positive (8) and negative (22, 23) influences on retronasal aroma; the stimulated salivary flow rate was presumed not to be substantially influenced by means of a simple correlation. However, multiple regression analysis showed the stimulated salivary flow rate was negatively correlated with aroma concentration. Saliva may require simultaneous application with chewing to affect the retronasal aroma. The effect of the dissolution of aroma in saliva may be stronger than the change in physical properties during chewing.

### Limitations

The sample size of this study was a small and the gender was only male. Since the masticatory performance varies depending on gender (36), further investigation with other genders is necessary. In addition, no consideration was given to the body constitution. There are several researches investigating the relationship between retronasal and obesity and body mass index (BMI) (5, 6, 37, 38). Although their association is often unclear, BMI and retronasal aroma can be interrelated factors. Larger sample sizes and adjustments are needed to investigate factors of the genders and the body constitution such as BMI and body composition.

The measurement of stimulated salivary flow rate in this experiment was not performed using saliva secreted during gummy jelly intake. The gummy jelly dissolves in saliva during chewing, which causes difficulty in correct measurement of saliva volume. Therefore, the analysis of stimulated salivary flow rate was performed using paraffin wax (26). The standardized gummy jelly is useful for assessment of natural chewing and quantitative evaluation of masticatory performance, but further analyses are needed to clarify its usage in studies of saliva.

In experiment 2, the swallowing suppression facilitated measurement of the increase in surface area after expectoration of the gummy jelly. Notably, this also suppressed stage II transport that propels the food bolus into the oropharynx (39) and the soft palatal movement that causes oropharyngeal opening (35). In addition, homogeneity and particle size of food bolus were altered by suppression of stage II transport (40). In this study, the aroma concentration was similar between free intake (with swallowing) and 100% of the swallowing threshold (without swallowing). Although the swallowing suppression may not have a strong effect on retronasal, these changes might have altered the masticatory and respiratory patterns, thereby affecting aroma release and transport. Measurement of respiration while chewing the gummy jelly may have helped to avoid effects on aroma release and transport. However, such assessment would have required the connection of another sensor to the nostril, which might have interfered with aroma measurement. Therefore, this respiratory measurement was not performed.

Despite the above limitations, to the best of our knowledge, this is the only study to simultaneously measure retronasal aroma and masticatory performance. Furthermore, the aroma concentration was correlated with the increase in surface area of gummy jelly, the number of chewing strokes, and the stimulated salivary flow rate. The findings of the present study suggested the presence of a new physiological state factor associated with retronasal aroma; this new physiological state factor is a combination of masticatory performance and number of chewing strokes. However, it should be noted that the aroma concentration was measured by the odor sensor and does not necessarily reflect the aroma perception. The relationships of masticatory function and retronasal aroma may also be associated with eating behaviors that cause obesity and metabolic syndrome; thus, further research is needed.

## Conclusions

Food aroma concentration, measured from the nostril while chewing the gummy jelly, varied depending on masticatory performance. In addition, the increase in surface area of gummy jelly and the number of chewing strokes were positively correlated with aroma concentration, while the stimulated salivary flow rate was negatively correlated with aroma concentration. These physiological mastication states are associated with retronasal aroma during food intake.

## Data Availability Statement

The original contributions presented in the study are included in the article/Supplementary Material, further inquiries can be directed to the corresponding author.

## Ethics Statement

The studies involving human participants were reviewed and approved by the ethics committee of the Faculty of Dentistry of Niigata University. The patients/participants provided their written informed consent to participate in this study.

## Author Contributions

This study was designed by JO and KH. The data were collected by JO and TY, and were analyzed by JO. The manuscript was drafted by JO and KH and was edited by TO. All authors contributed to the article and approved the submitted version.

## Conflict of Interest

The authors declare that the research was conducted in the absence of any commercial or financial relationships that could be construed as a potential conflict of interest.
