# Supplementary figures and images for: Higher Masticatory Performance and Higher Number of Chewing Strokes Increase Retronasal Aroma
Source: Front Nutr. 2021 Mar 2;8:623507. doi: 10.3389/fnut.2021.623507 (PMC7960666; doi:10.3389/fnut.2021.623507)

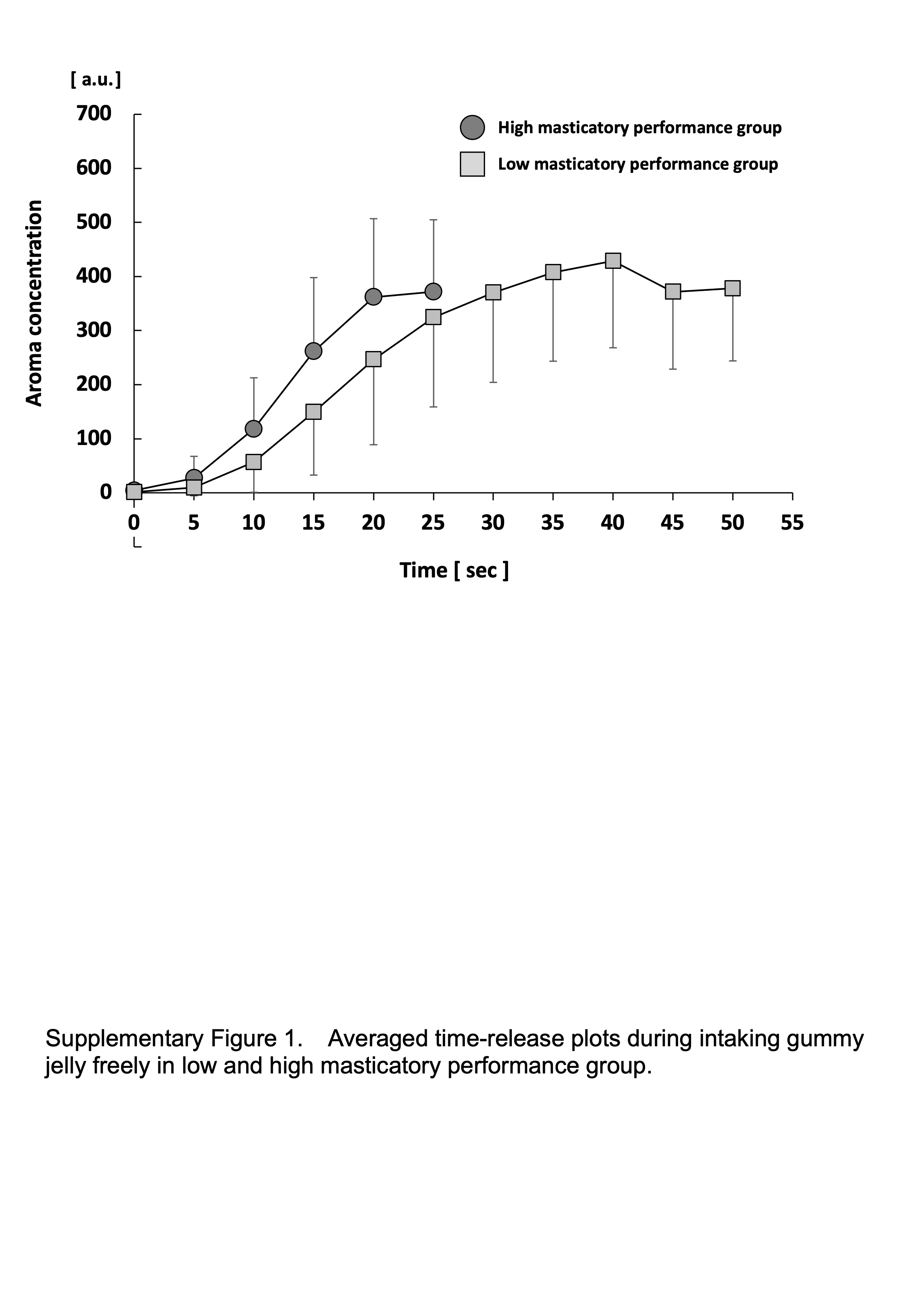

Supplement: Supplementary file 1 [file Image_1.TIFF]
